# Amifostine (WR-2721) Mitigates Cognitive Injury Induced by Heavy Ion Radiation in Male Mice and Alters Behavior and Brain Connectivity

**DOI:** 10.3389/fphys.2021.770502

**Published:** 2021-11-16

**Authors:** Sydney Weber Boutros, Benjamin Zimmerman, Sydney C. Nagy, Joanne S. Lee, Ruby Perez, Jacob Raber

**Affiliations:** ^1^Department of Behavioral Neuroscience, Oregon Health & Science University, Portland, OR, United States; ^2^Advanced Imaging Research Center, Oregon Health & Science University, Portland, OR, United States; ^3^Beckman Institute for Advanced Science and Technology, University of Illinois at Urbana-Champaign, Urbana, IL, United States; ^4^Departments of Neurology and Radiation Medicine, Oregon Health & Science University, Portland, OR, United States; ^5^Division of Neuroscience, Oregon National Primate Research Center, Portland, OR, United States

**Keywords:** amifostine, heavy ion radiation, cognition, cFos, sex differences

## Abstract

The deep space environment contains many risks to astronauts during space missions, such as galactic cosmic rays (GCRs) comprised of naturally occurring heavy ions. Heavy ion radiation is increasingly being used in cancer therapy, including novel regimens involving carbon therapy. Previous investigations involving simulated space radiation have indicated a host of detrimental cognitive and behavioral effects. Therefore, there is an increasing need to counteract these deleterious effects of heavy ion radiation. Here, we assessed the ability of amifostine to mitigate cognitive injury induced by simulated GCRs in C57Bl/6J male and female mice. Six-month-old mice received an intraperitoneal injection of saline, 107 mg/kg, or 214 mg/kg of amifostine 1 h prior to exposure to a simplified five-ion radiation (protons, ^28^Si, ^4^He, ^16^O, and ^56^Fe) at 500 mGy or sham radiation. Mice were behaviorally tested 2–3 months later. Male mice that received saline and radiation exposure failed to show novel object recognition, which was reversed by both doses of amifostine. Conversely, female mice that received saline and radiation exposure displayed intact object recognition, but those that received amifostine prior to radiation did not. Amifostine and radiation also had distinct effects on males and females in the open field, with amifostine affecting distance moved over time in both sexes, and radiation affecting time spent in the center in females only. Whole-brain analysis of cFos immunoreactivity in male mice indicated that amifostine and radiation altered regional connectivity in areas involved in novel object recognition. These data support that amifostine has potential as a countermeasure against cognitive injury following proton and heavy ion irradiation in males.

## Introduction

The possibility of extended human space travel is getting closer and closer to fruition. An important factor to consider is the safety of flight teams on extended missions, such as to the moon and to Mars. Galactic cosmic rays (GCRs) and solar particle events (SPEs) are unique and dangerous features of space travel. GCRs are comprised of ionized atomic nuclei from naturally occurring elements, such as hydrogen and silicon, while SPEs primarily contain low-to-medium energy protons (Simonsen et al., 2020). Both pose a risk to astronauts during and following missions, in addition to the other physical and psychological strains that are inherent to deep-space flights (Stuster, 2010). Moreover, recent cancer therapies are utilizing protons and heavier ions (Pompos et al., 2016). Proton radiotherapy decreases damage to healthy tissue and is overall associated with higher survival rates (Higgins et al., 2017).

Previous animal research supported by NASA involved studying the effects of single heavy ion exposure on behavioral and cognitive performance, with a specific focus on hippocampal function. Studies from our lab and others have shown altered hippocampal function following exposure to particles present in the space environment such as protons (Sweet et al., 2014; Parihar et al., 2015; Sokolova et al., 2015; Impey et al., 2016b; Rudobeck et al., 2017), ^16^O (Poulose et al., 2011; Raber et al., 2015a; Rabin et al., 2015), ^56^Fe ions (Shukitt-Hale et al., 2003; Rola et al., 2004; Villasana et al., 2008; Vlkolinský et al., 2008; Allen et al., 2015), and ^28^Si ions (Raber et al., 2015b; Whoolery et al., 2017). These studies uphold early work from the Soviet space program: male Wistar rats that were exposed to 24-h gamma radiation on the Cosmos 690 satellite in 1974 showed impaired spatial navigation and decreased ability to handle mental workloads (Ahlers et al., 1976; Clement et al., 2020).

Investigations into the effects of combinations of charged particles have only more recently been pursued. For example, we reported earlier that novel object recognition was impaired in male and female mice 3 months following 500 and 2000 mGy doses of a combination of three beams (protons, ^16^O, and ^28^Si), and neuronal inflammatory markers differed between the sexes (Raber et al., 2019). Similarly, female mice that received a dose of 500 or 2000 mGy six beam radiation (protons, ^4^H, ^16^O, ^28^Si, ^48^Ti, and ^56^Fe) also showed novel object recognition impairment 3 months later, and both males and females expressed fear memory impairment (Raber et al., 2020).

Exposure to both individual and combined particles has long-term effects on the central nervous system. We have previously shown that simulated space radiation with protons or ^56^Fe ions alters expression of immediate-early genes (IEGs), specifically Arc mRNA levels (Raber et al., 2014, 2016; Impey et al., 2016a,^2017^). IEGs are essential for synaptic plasticity and play an important role in learning and memory (Richardson et al., 1992; Demmer et al., 1993; Yin et al., 2002). Hippocampal cFos expression specifically has been shown to be essential for spatial learning and memory, including spatial habituation and novel object recognition (Barbosa et al., 2013). It is possible, then, that alterations in IEGs due to space radiation contribute to the observed behavioral and cognitive impairments. Moreover, it is likely that there are brain-wide disruptions, but limitations in technology have restricted wide-scale whole brain network changes.

Beyond the hippocampus, several other brain regions have been identified as important for intact object recognition, such as the sensorimotor cortex, amygdala, rhinal cortex, and subiculum (Moses et al., 2005; Antunes and Biala, 2012; Chen Y. et al., 2018). Recent advances in whole-brain immunohistochemistry and microscopy have opened up the ability to assess cFos expression throughout the brain (Kim et al., 2015, 2017; Renier et al., 2016), providing unique opportunities to easily analyze many regions essential for specific tasks as well as explore regional connectivity (Zuloaga et al., 2015).

Considering the evidence pointing to detrimental cognitive effects following simulated single particle- and combined-GCR exposure, it is important to develop strategies to mitigate these effects. Amifostine (WR-2721) is an FDA-approved radioprotectant commonly used during cancer treatment to protect non-tumorous tissue from photon radiation (Bensadoun et al., 2006; Dziegielewski et al., 2008; Gu et al., 2014; Buntzel et al., 2016). It is cleaved into the active metabolite WR-1065, which protect cells by scavenging free radicals, increasing the speed of DNA repair, and mitigating other immune signals (Dziegielewski et al., 2008). In male mice, amifostine (214 mg/kg, 30 min before exposure to 2 Gy of ^60^Co gamma-rays at dose-rate of 3.1 Gy/min) mitigated the effects of gamma radiation on novel object recognition 2 days after exposure (Lee et al., 2010). Our preliminary data also indicated that an amifostine analog administered once prior to exposure with ^28^Si ions (0.2 Gy, 300 MeV/n) had long-term effects on novel object recognition 3 months following exposure, but that these effects were dependent on sex: irradiated males showed cognitive impairment that was rescued by the amifostine analog, but this did not change behavior in females (Bacher et al., 2019). Research into the effects of amifostine in females is glaringly lacking, though. Females clear plasma amifostine faster than males (McKibbin et al., 2010). However, to the best of our knowledge, there are no reports of the effects of amifostine on learning and memory outcomes in females.

Here, we tested if a single treatment with amifostine given prior to radiation exposure could mitigate the long-term behavioral alterations and cognitive deficits induced by space radiation using 6-month-old male and female C57Bl/6J mice. We predicted that both a middle (107 mg/kg) and high (214 mg/kg) dose of amifostine would reduce behavioral alterations and rescue cognitive deficits in male and female mice 3 months following a simplified five-beam exposure. Additionally, we sent a sub-set of brains to Certerra, Inc. for whole-brain imaging of cFos to assess the effects of radiation and amifostine on IEG expression. We hypothesized that exposure to the simplified five-beam would alter cFos expression and that amifostine would normalize expression. As – to the best of our knowledge – this is the first study that assesses the effects of amifostine in both sexes, we did not originally predict different effects in males and females. Our results can inform novel cancer therapies as well as countermeasures that can be taken by astronauts on deep-space missions. While these two populations – cancer patients and astronauts – are notably distinct, research characterizing exposure to heavy ions and mitigating factors is informative for both groups.

## Materials and Methods

### Mice and Radiation Exposure

Ninety-six C57Bl/6J (WT) male and female mice were ordered from Jackson Laboratories (Bar Harbor, ME) at 6 months of age. Mice were delivered to and housed in the NASA Space Radiational Laboratory (NSRL) at the Brookhaven National Laboratory (BNL). Two weeks after acclimating to the BNL animal facilities, mice were exposed to a whole-body 500 mGy dose of simplified five-beam GCR (Simonsen et al., 2020) or a sham exposure. The simplified five-beam GCR is delivered sequentially as follows: protons, ^28^Si, ^4^He, ^16^O, and ^56^Fe, protons (for dose fractions and energies, see Table 1). One hour prior to radiation or sham exposure, mice were treated with an intraperitoneal (i.p.) injection of saline, 107 mg/kg, or 214 mg/kg of amifostine. These doses were chosen as they have previously been shown to counter negative effects of whole-body gamma radiation (Lee et al., 2010; Cheema et al., 2019). One week later, mice were shipped to the Oregon Health & Science University (OHSU). Behavioral and cognitive testing occurred 2–3 months after exposure, as our main goal was to assess long-term effects. Estrous cycle was not tracked in females.

**TABLE 1:** Break-down of the simplified five-beam radiation components.

Ion species	Energy (MeV/n)	LET	Dose (mGy)	Dose fraction
Proton	1000	0.2	174.1	0.35
^28^Si	600	50.4	5.7	0.01
^4^He	250	1.6	90.2	0.18
^16^O	350	20.9	29.1	0.06
^56^Fe	600	173.8	5.1	0.01
Proton	250	0.4	195.9	0.39

**FIGURE 1:**
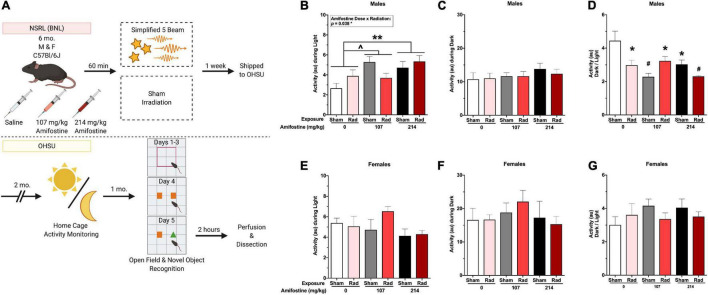
Experimental timeline and home-cage activity data. **(A)** Six-month-old male and female C57Bl/6J mice received an injection of saline, 107 mg/kg, or 214 mg/kg of amifostine 60 min before exposure to the simplified five-beam simulated galactic cosmic rays or the sham hotel. Mice were then shipped to the authors’ institution. Two months following exposure, home-cage activity was recorded for 1 week. After another month, mice underwent open field and novel object recognition tests. Two hours following the novel object test, mice were euthanized, and tissue collected. **(B–G)** Home cage activity data. In male mice, we found a main effect of amifostine (*p* = 0.010) and amifostine-by-radiation interaction (*p* = 0.038) during the light cycle **(B)**. Mice injected with saline were significantly different than mice injected with 214 mg/kg (*p* = 0.005) and trended toward different from 107 mg/kg (*p* = 0.054). No differences in average dark activity were found in males **(C)**. There was an effect of amifostine (*p* = 0.003) and amifostine-by-radiation interaction (*p* = 0.002) when the ratio of activity during the dark to activity during the light was analyzed in male mice **(D)**. All groups were different than the Sham-Saline group. In female mice, there were no differences in average light activity **(E)**, average dark activity **(F)**, or the ratio **(G)**. Data are presented as means ± SEMs. ^∧^*p* < 0.06; *p < 0.05; **p < 0.01; ^#^*p* < 0.001.

Animals were group housed 4 to a cage throughout the duration of this study, except during the 1-week activity monitoring period (see below). Food and water were provided *ad libitum*. Lights were on a standard 12 h light: dark cycle. Mice were split evenly between groups, such that *n* = 8 mice per sex per amifostine dose per radiation condition. Animals were monitored daily by staff for signs of pain or distress. Body weights were recorded weekly over the entire course of the experiment, starting prior to radiation or sham exposure. The entire experimental design is depicted in Figure 1A.

All animal procedures were consistent with ARRIVE guidelines and reviewed and approved by the Institutional Animal Care and Use Committee at BNL and OHSU.

### Activity Monitoring

To assess the effects of heavy ion exposure and amifostine on sleep-wake cycles, animals were placed into non-invasive home cage activity monitoring devices 2 months after exposure to sham or radiation (MLog, Biobserve, Germany) for 1 week (Johnson et al., 2015). Motion was recorded every 1 s in arbitrary units (au). Throughout monitoring, mice were singly housed and provided with extra nest-building material. As we have only 24 cameras, mice were split into 4 cohorts for activity monitoring, over the course of 1 month. All groups were represented in each cohort to control for cohort effects and time post-exposure. As each cohort was in activity monitors over the course of a week, the entire estrus cycle of the female mice was captured (Ajayi and Akhigbe, 2020).

### Open Field and Novel Object Recognition

To assess the long-term effects of heavy ion exposure and amifostine, mice were tested for anxiety-like behavior and spatial habituation learning in the open field and for novel object recognition memory 3 months following exposure, as described (Raber et al., 2019). For the open field, mice were placed into a 41 cm × 41 cm chamber for 5 min over three consecutive days. The center area was defined as the 20 cm × 20 cm square in the center of the open field. Total distance moved, velocity, and time spent in the center area of the open field were recorded and analyzed.

On the fourth day, two identical orange octagon blocks were placed within the center area of the arena. The next day, one object was replaced with a distinct, novel object (a green triangle block). Both trials were 15 min. Time spent with the familiar and novel objects was analyzed to assess hippocampus-dependent memory. Light for all open field and novel object trials was at 100 lux.

Behavioral performance during the open field and object recognition tests were video recorded and data exported with Ethovision vs. 14.1 software (Wageningen, Netherlands). Arenas and objects were thoroughly cleaned with 0.5% acetic acid between trials.

### Tissue Collection

Two hours following the novel object recognition test, mice were euthanized to capture peak cFos expression (Zhu et al., 2010). Males were euthanized by perfusion: briefly, mice were deeply anesthetized with a 50 mg/kg ketamine–xylazine cocktail and perfused with ice-cold saline followed by 4% paraformaldehyde (PFA). Brains were removed and stored overnight in 4% PFA, then transferred to saline. Based on behavioral results, we selected to send brains from all males in the 0 and 107 mg/kg, radiation and sham groups to Certerra, Inc. (Farmingdale, NY, United States) for whole brain staining and imaging of cFos expression.

Females were euthanized by cervical dislocation and rapid decapitation 2 h after completing novel object recognition. Hippocampus and cortex were dissected, and flash frozen in liquid nitrogen, then stored at −80°C for future use, not reported in this current study.

### Whole Brain cFos Imaging and Analysis

Whole brain cFos staining and light sheet imaging was performed by Certerra, Inc. (Farmingdale, NY, United States), as previously described (Kim et al., 2015). Based on the behavioral data, we selected four groups of males for analysis: Sham-Saline, Radiation-Saline, Sham-107 mg/kg Amifostine, and Radiation-107 mg/kg Amifostine (*n* = 8 mice/condition). These groups were chosen as we wanted to assess the lowest effective dose tested. The data generated were in the form of raw number of cFos+ cells within each brain region defined by the Allen Mouse Brain Atlas.

### Statistics

All data were first assessed for homogeneity to confirm use of standard parametric tests. Data were analyzed using SPSS vs. 25 (IBM, Armonk, NY, United States) and GraphPad vs. 7 (Prism, San Diego, CA, United States).

All analyses were first performed as multi-way ANOVAs with sex, amifostine dose, and radiation exposure as between group variables. As we repeatedly found significant effects and interactions with sex, we proceeded to split sexes for analysis to clarify the effects of radiation and amifostine on our measures. The statistical results with sex as a variable can be seen in Supplementary Table 1.

For body weight, a two-way ANOVA was used with radiation exposure and amifostine dose as between-group variables.

For activity monitoring, a repeated measures ANOVA was used with time as a within-group variable and radiation exposure and amifostine dose as between-group variables. Activity over the course of the light periods was analyzed separately from the dark periods.

For the open field, a repeated measures ANOVA was used with trial as a within-group variable and exposure and amifostine dose as between-group variables. Total distance moved (cm), average velocity (cm/s), and percent time in the center were analyzed. For object recognition, time spent with the objects was analyzed using a two-way ANOVA on day 1 and day 2. Prior to analyzing hippocampus-dependent memory in the novel object recognition test, any mice that explored <2 s on either day 1 or day 2 were removed. To assess memory, percent time spent exploring the objects was calculated, and paired samples *t*-tests used to compare the familiar vs. the novel object within each group. A discrimination index (DI) was then calculated by subtracting time exploring the familiar object from time exploring novel object, and then dividing this difference by the total time spent exploring both objects (Antunes and Biala, 2012). Written as a formula,


$$DI=\left(T_{N}-T_{F}\right)÷T_{T}$$

T_*N*_ is time with novel object (s), T_*F*_ is time with familiar object (s), and T_*T*_ is total time spent exploring the objects. The DI measure was then analyzed using a two-way ANOVA.

Following all ANOVAs, *post hoc* analyses were used to assess groups compared to the control group (Sham-Saline), as statisticians have indicated that *post hoc* analyses are acceptable in the absence of significant ANOVAs (Chen T. et al., 2018).

While data indicated that standard parametric tests could be employed for whole brain analysis, initial assessment of cFos immunoreactivity in specific brain regions using a general linear model Poisson regression indicated that goodness of fit was poor. Thus, we proceeded to analyze the raw data using a negative binomial regression across the whole brain and within brain regions important for novel object recognition (Antunes and Biala, 2012). A list of identified brain regions and their contribution to novel object recognition can be seen in Supplementary Table 3.

Due to the unique features of whole-brain data, we assessed the relationship of cFos immunoreactivity signal across related brain regions within individual mice as an indirect measure of connectivity, similar to other analyses of cFos immunoreactivity in coronal sections (Zuloaga et al., 2015). We first took an unbiased approach to look at connectivity across the cerebrum, brainstem, and cerebellum, followed by a defined approach to assess connectivity across regions important for the 24 h novel object recognition test. Distinct Pearson’s correlations were run for brain regions in the following groupings: cerebrum, brainstem, cerebellum, and regions associated with novel object recognition (Antunes and Biala, 2012). Correlation matrices were created from the *r* values. The matrices from each group were then compared using the High Dimensional Test (HD Test) for Mean Vectors, Covariance Matrices, and White Noise of Vector Time series (Cai et al., 2014). Statistical analyses for comparing correlation matrices were performed using R 4.0.3 (R Development Core Team, 2020), specifically with the xlsx (Dragulescu and Arendt, 2020) and the HD Test (Cao et al., 2018) packages. Correlation matrices were compared using a method developed for testing the equality of covariance matrices when the dimensionality of the covariance matrix is larger than the sample size (Chang et al., 2017). Across all experimental groups and defined regional groupings, we ran a total of 24 comparisons; as such, we used Bonferroni’s *post hoc* correction.

To integrate dependent variables, we performed a principal component analysis (PCA) to determine how amifostine or radiation may affect the relationship between different measures (Pfankuch et al., 2005). We included the following variables in the PCA: activity during the light, activity during the dark, ratio of activity during the light to the dark, total distance moved on day 1 of the open field, percent time spent in the center on day 1 in the open field, the difference in total distance moved between day 1 and day 2 in the open field, the difference in total distance moved between day 2 and day 3 in the open field, the difference in percent time in the center between day 1 and day 2 in the open field, the difference in percent time in the center between day 2 and day 3 in the open field, the total time exploring the objects during both days of the novel object test, and the percent time spent with the novel object on test day. As we detected significant effects between males and females in our original PCA, we ran PCAs separately in each sex. Following the PCA, we used an ANOVA to compare the reduction scores across radiation and amifostine doses.

## Results

### Male and Female C57Bl/6J Mice Show Differences at Baseline and in Response to Amifostine and Radiation

A schematic of the experimental design can be seen in Figure 1A. The break-down of radiation exposure can be seen in Table 1.

We first assessed the effects of amifostine and radiation with sex as a between-subject variable. Throughout our measures, we found both baseline differences between males and females, as well as differing reactions to amifostine and radiation, indicated by statistical interactions. A list of the sex effects and interactions in our behavioral and cognitive measures is indicated in Supplementary Table 1. Due to the statistical interactions, we proceeded to analyze male and female data separately to provide more clarity.

### Amifostine and Radiation Both Alter Activity During the Light Cycle in Male, but Not Female, C57Bl/6J Mice

Neither amifostine nor radiation changed body weight (Supplementary Figure 1).

All mice showed higher activity during the dark period compared to the light period (Figures 1B,C,E,F). Analysis of the average activity over the course of a week during the light periods revealed a significant main effect of amifostine dose (*F*_2,42_ = 5.134, *p* = 0.009) and a significant radiation-by-amifostine dose interaction (*F*_2,42_ = 3.673, *p* = 0.034) in males (Figure 1B). Dunnett’s *post hoc* indicated that mice injected with 214 mg/kg of amifostine moved more during the light than saline-treated mice (*p* = 0.005) and mice injected with 107 mg/kg trended toward moving more than the saline-treated mice (*p* = 0.054). There were no significant differences in activity during the dark period (Figure 1C). In contrast to the males, there were no significant differences in activity in either light or dark period detected in females (Figures 1E,F).

The ratio of activity during the dark period to the light period was analyzed to measure the disruption to typical activity rhythms. We detected a significant main effect of amifostine dose (*F*_2,42_ = 6.650, *p* = 0.003) and a significant radiation-by-amifostine dose interaction (*F*_2,42_ = 7.618, *p* = 0.002) in males (Figure 1D). Dunnett’s *post hoc* indicated that all radiation and/or amifostine-treated male groups were significantly different than the control male group. In contrast, no significant differences were detected in the ratio activity measure in females (Figure 1G).

### Amifostine and Radiation Affect Spatial Habituation and Anxiety-Like Measures in a Sex-Dependent Manner

**FIGURE 2:**
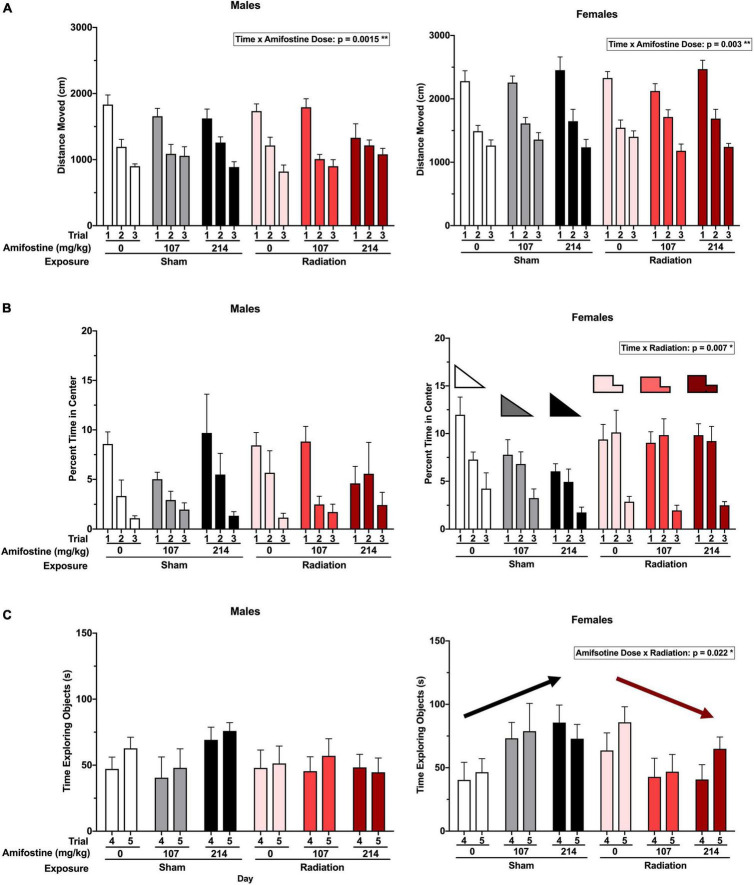
Activity and anxiety-like measures in the open field and novel object tests. **(A)** The total distance moved (cm) in open field in males (*left*) and females (*right*). A time by amifostine interaction was found in males (*p* = 0.0015) and females (*p* = 0.003). **(B)** Percent time in the center of the open field in males (*left*) and females (*right*). Females showed a time by radiation interaction (*p* = 0.007). **(C)** Total time exploring objects over the training and testing day in males (*left*) and females (*right*). Females showed an amifostine by radiation interaction (*p* = 0.022). Data are presented as means ± SEMs.

Hippocampus-dependent spatial habituation was assessed using total distance moved over 3 days in the open field. Analysis indicated a significant time-by-amifostine dose interaction in both males (*F*_3.076,64.592_ = 3.736, *p* = 0.015) and females (*F*_4,84_ = 4.337, *p* = 0.003; Figure 2A). In males, this interaction was driven by a distinct pattern of change over the 3 days in mice injected with 107 mg/kg of amifostine, where there was almost no decrease between day 2 and day 3. Additionally, the males injected with 214 mg/kg of amifostine showed a blunted decrease in activity over the 3 days; effects of both these doses differed from the expected large decreases seen in the saline-injected males. In females, the time-by-amifostine interaction was primarily driven by the irradiated mice injected with the 107 mg/kg dose showing a larger change in activity between day 2 and day 3.

Anxiety-like behavior was also assessed by analyzing the time spent in the more anxiety-provoking center of the open field. There were no differences detected based on radiation or amifostine in male mice (Figure 2B). Conversely, a repeated measures ANOVA indicated a significant time-by-exposure interaction in females (*F*_2,84_ = 5.310, *p* = 0.007; Figure 2B). Female mice exposed to radiation in the absence or presence of amifostine did not show the expected decrease in time spent in the center of the open field on day 2.

Similarly, object exploration was altered in female, but not male, mice. We detected an exposure-by-amifostine dose interaction (*F*_2,42_ = 4.195, *p* = 0.022) in females only (Figure 2C). Sham-irradiated female mice that were injected with amifostine explored the objects more on both days of the object recognition test than vehicle-treated sham-irradiated mice; conversely, irradiated females that received amifostine explored the objects less than vehicle-treated irradiated mice.

### Amifostine Mitigates Radiation-Induced Cognitive Impairment in Males, but Amifostine and Radiation Combined Impair Cognition in Females

**FIGURE 3:**
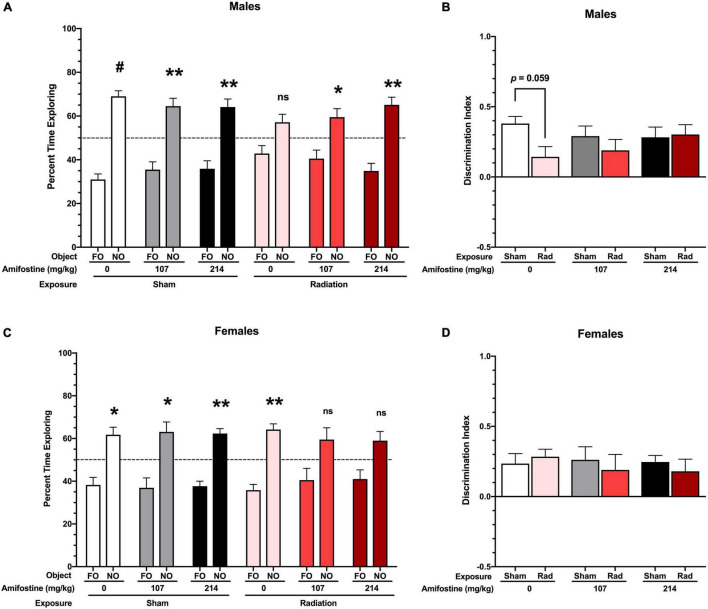
Performance in the novel object recognition test. **(A)** Percent time spent with the familiar and novel object in male mice. All sham-exposed male mice showed a preference for the novel object (Sham-Saline: *p* < 0.001; Sham-107 mg/kg: *p* = 0.007; and Sham-214 mg/kg: *p* = 0.006). Mice exposed to radiation did not show a preference (*p* = 0.09); however, both 107 and 214 mg/kg of amifostine restored preference for the novel object (*p* = 0.045 and *p* = 0.003, respectively). **(B)** Discrimination index (DI) in male mice. A trend toward a difference based on radiation exposure (*p* = 0.071) was found, and Sidak’s *post hoc* test revealed a trend toward a decrease DI in the Rad-Saline group compared to Sham-Saline (*p* = 0.0589). **(C)** Percent time spent with the familiar and novel object in female mice. All mice in the sham exposure groups showed a preference for the novel object (Sham-Saline: *p* = 0.021; Sham-107 mg/kg: *p* = 0.026; and Sham-214 mg/kg: *p* = 0.001). Female mice exposed to radiation also showed a preference for the novel object (*p* = 0.001), but females that received 107 or 214 mg/kg of amifostine prior to radiation did not show a significant preference (*p* = 0.137 and *p* = 0.075, respectively). **(D)** DI in female mice. No significant differences were detected. Data are presented as means ± SEMs. *p < 0.05; **p < 0.01; ^#^*p* < 0.001.

All sham-irradiated mice showed a significant preference for exploring the novel object. However, male mice exposed to radiation failed to show a significant preference for the novel object. Pre-treatment with both doses of amifostine restored preference for the novel object, though (Figure 3A). To directly compare performance across groups, we used the DI measure. This revealed a trend toward a significant main effect of exposure (*F*_1,41_ = 3.432, *p* = 0.071); Sidak’s *post hoc* test identified a trend toward a significant difference in the Sham-Saline compared to the Rad-Saline group (*p* = 0.059; Figure 3B). There were no differences detected between the sham and irradiated groups that received amifostine injections.

Female sham-irradiated mice also showed in-tact hippocampus-dependent memory, with all sham-irradiated groups displaying a significant preference for the novel object. Unlike the male mice, the irradiated female mice that received saline injections showed a preference for the novel object, while the irradiated females that received amifostine pre-treatment failed to show a preference (Figure 3C). A two-way ANOVA did not indicate any significant differences in the DI measure in females (Figure 3D).

### Radiation and 107 mg/kg of Amifostine Increase Co-activation Across the Cerebrum, Brainstem, and in Regions Associated With Novel Object Recognition in Male Mice

We first analyzed cellular activation using a negative bimodal regression of cFos immunoreactivity across the brain in the 4 groups included: Sham-Saline, Sham-107 mg/kg, Rad-Saline, and Rad-107 mg/kg. These groups were chosen based on the novel object recognition data, as we wanted to analyze the lowest dose of amifostine that changed behavior. Neither radiation nor 107 mg/kg of amifostine altered the number of cFos+ cells (Supplementary Figure 2), indicating that neither radiation nor drug treatment changed the global magnitude of cellular activation as measured by cFos immunoreactivity.

**FIGURE 4:**
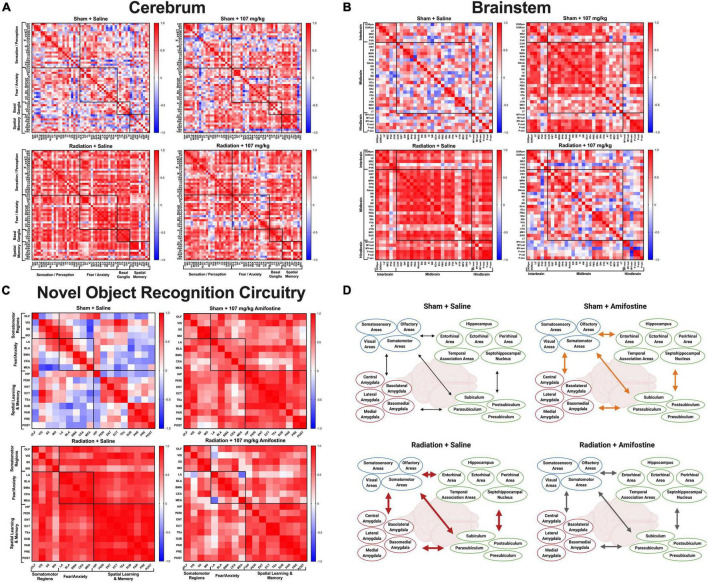
Whole brain cFos data. Pearson’s correlations were run for all regions in each distinct group: Sham-Saline (*top left*), Sham-107 mg/kg (*top right*), Rad-Saline (*bottom left*), and Rad-107 mg/kg (*bottom right*). Bonferroni correction was applied to comparisons of the correlation matrices. **(A)** cFos connectivity in the cerebrum. All groups were significantly different from each other. **(B)** cFos connectivity in the brainstem. All groups were significantly different from each other. **(C)** cFos connectivity in regions important for 24 h novel object recognition. Most groups were significantly different from each other. No difference was detected between Sham-107 mg/kg and Rad-Saline (*p* = 0.0912). Full names for brain regions can be seen in Supplementary Tables 2, 3. **(D)** Schematic depicting the changes in co-activation of brain regions important for novel object recognition. Both radiation and amifostine increased the correlations between brain regions. Radiation appeared to cause strong, positive correlations across all brain regions (depicted by thick, red arrows); amifostine appeared to do the same, albeit to a lesser extent (depicted by thick, orange arrows). Radiation +107 mg/kg amifostine somewhat ameliorated the strong, positive correlations induced by radiation, though the correlations were still stronger than the Sham-Saline group (depicted by medium, gray arrows). Created with BioRender.com.

With this measure of cellular activation, we were able to compare the co-activation of brain regions in each group using correlation matrices. When we analyzed connectivity in the cerebrum, we discovered that all groups were distinct. The correlation matrix of the control group (Sham-Saline) was different from the Sham-107 mg/kg (χ^2^ = 7.76, *p* < 0.0001), Rad-Saline (χ^2^ = 8.37, *p* < 0.0001), and Rad-107 mg/kg (χ^2^ = 8.51, *p* < 0.0001; Figure 4A) groups. Both radiation and amifostine treatment increased the correlations between regions compared to the Sham-Saline group. Our analysis also revealed that the Rad-Saline group was significantly different from the Rad-107 mg/kg (χ^2^ = 6.56, *p* = 0.0432) and the Sham-107 mg/kg (χ^2^ = 6.78, *p* < 0.0001) groups, such that the Rad-Saline group had an increase in regional correlations compared to the amifostine-treated groups. Lastly, comparison of the correlation matrices for the Rad-107 mg/kg group and the Sham-107 mg/kg group also revealed these groups to be significantly different (χ^2^ = 7.08, *p* < 0.0001) with the Rad-107 mg/kg showing an increase in regional correlation.

We similarly assessed connectivity in the brainstem. As with the cerebrum, we found that the correlation matrix of the Sham-Saline control group was significantly different than the Sham-107 mg/kg group (χ^2^ = 6.38, *p* < 0.0001), Rad-Saline group (χ^2^ = 5.71, *p* < 0.0001), and Rad-107 mg/kg group (χ^2^ = 7.76, *p* < 0.0001; Figure 4B), again in the direction that radiation and amifostine increased regional correlations. The Rad-Saline and the Rad-107 mg/kg groups were also different from each other (χ^2^ = 6.51, *p* < 0.0001), with amifostine appearing to lead to an overall decrease in the amount of strong, positive correlations observed in the Rad-Saline group. The Sham-107 mg/kg group was also significantly different from the Rad-107 mg/kg group (χ^2^ = 6.64, *p* < 0.0001), though the Sham-107 mg/kg group had more strongly positive correlations than the Rad-107 mg/kg group. Comparing the Rad-Saline and the Sham-107 mg/kg groups, there was a trend toward a difference, but it did not reach significance (χ^2^ = 4.37, *p* = 0.058).

To complete the unbiased connectivity analysis, we compared the correlation matrices of regions in the cerebellum. We did not detect differences when we compared the Sham-Saline group to Sham-107 mg/kg (χ^2^ = 3.22, *p* = 0.370), Rad-0 mg/kg (χ^2^ = 3.22, *p* = 0.389), or Rad-107 mg/kg (χ^2^ = 3.23, *p* = 0.518, Supplementary Figures 3A–D). Similarly, there were no differences between the Sham-107 mg/kg and either group exposed to radiation (Rad-Saline: χ^2^ = 3.56, *p* = 0.322; Rad-107 mg/kg: χ^2^ = 4.07, *p* = 0.0912). We did detect a difference in the Rad-107 mg/kg compared to the Rad-Saline group, though (χ^2^ = 4.65, *p* = 0.0048), suggesting that amifostine modulated effects of radiation in the cerebellum as well. A breakdown of all the regions used for connectivity analysis of the cerebrum, brainstem, and cerebellum is indicated in Supplementary Table 2.

We next analyzed brain activation in regions known to play a role in 24-h novel object recognition (Supplementary Table 3). Similar to our findings with the unbiased whole brain analysis, radiation and amifostine did not significantly affect the number of cFos+ cells in distinct brain regions (Supplementary Figure 4). Analysis of the correlation matrices indicated that the sham-irradiated, vehicle-treated control group was significantly different from the Sham-107 mg/kg group (χ^2^ = 5.68, *p* < 0.0001), Rad-Saline group (χ^2^ = 5.29, *p* < 0.0001), and Rad-107 mg/kg group (χ^2^ = 5.92, *p* < 0.0001; Figure 4C). Both radiation and amifostine appeared to increase the connectivity in the task-specific network. Comparison of the experimental groups revealed that the Sham-107 mg/kg group was significantly different from the Rad-107 mg/kg group (χ^2^ = 4.762, *p* = 0.0048), but not the Rad-Saline group (χ^2^ = 3.97, *p* = 0.0912). Notably, the Rad-Saline and Rad-107 mg/kg groups were significantly different from each other (χ^2^ = 4.182, *p* = 0.0432), with amifostine treatment attenuating the correlations between regions. An overall schematic depicting the effects of radiation and amifostine on connectivity in regions necessary for novel object recognition analyzed by cFos immunoreactivity is illustrated in Figure 4D. The raw data for the whole-brain analysis can be found in Supplementary File 1.

### Principle Components Analysis Reveals Data Variance Is Different Between Males and Females

When a PCA was performed, five reduction factors were identified. The first factor accounted for 21.86% of the variance, the second for 19.02%, the third for 13.78%, the fourth for 11.87%, and the fifth for 9.69%. An ANOVA to explore possible differences based on sex, amifostine dose, or radiation indicated that factor 1 and factor 3 were different between males and females (*p* < 0.001 and *p* = 0.002, respectively). Additionally, an amifostine dose by sex interaction was detected for factor 1 (*p* = 0.027), factor 2 (*p* = 0.013), and factor 3 (*p* = 0.038), as well as an amifostine by radiation by sex interaction for factor 1 (*p* = 0.043). The loadings for the factors and the variables in each are indicated in Table 2.

**TABLE 2:** Results from PCA in female and male mice.

	Sexes collapsed	Percent (%) variance	Females	Percent (%) variance	Males	Percent variance
Component 1		**21.86**		**28.17**		**25.85**
	TotDist_OF1	84.40	Activity_Light	85.90	Diff-TotDist1–2	87.50
	Diff-TotDist1–2	69.80	Activity_Dark	82.80	TotDist_OF1	77.70
	Activity_Ratio	68.40	Diff-TotDist2–3	64.50	Diff-PctCenter1–2	74.50
	Activity_Dark	38.10	Diff-PctCenter2–3	60.40	PctCenter_OF1	65.30
			TotDist_OF1	41.10		
Component 2		**19.02**		**18.97**		**17.60**
	Diff-TotDist2–3	74.40	Diff-TotDist1–2	84.60	Activity_Light	95.60
	Diff-PctCenter2–3	67.70	TotDist_OF1	79.80	Activity_Dark	74.50
			PctTime_Novel	60.40		
Component 3		**13.78**		**15.75**		**15.12**
	Activity_Light	95.40	Diff-PctCenter1–2	82.00	Diff-PctCenter2–3	92.10
	Activity_Dark	77.20	PctCenter_OF1	79.30	PctCenter_OF1	54.90
			TimeExploringObjs_Day1–2	48.30	Diff-TotDist2–3	44.20
Component 4		11.87		**10.83**		**11.21**
	PctCenter_OF1	93.50	Activity_Ratio	89.60	PctTime_Novel	75.80
	Diff-PctCenter2–3	53.90	TimeExploringObjs_Day1–2	46.40	TimeExploringObjs_Day1–2	65.20
	TimeExploringObjs_Day1–2	40.50	Diff-PctCenter2–3	36.70		
	Diff-PctCenter1–2	40.10				
Component 5		**9.69**	x		x	
	PctTime_Novel	81.90				
	TimeExploringObjs_Day1–2	68.00				

*Data abbreviations as follows: Activity_Light, activity during the light cycle in home cage activity monitoring; Activity_Dark, activity during the dark cycle in home cage activity monitoring; TotDist_OF1, total distance moved during day 1 of open field; Diff-TotDist1–2, difference in total distance moved between day 1 and day 2 of open field; Diff-TotDist2–3, difference in total distance moved between day 2 and day 3 of open field; PctCenter_OF1, percent time spent in the center of the open field on day 1; Diff-PctCenter1–2, difference in time spent in the center of the open field between day 1 and day 2; Diff-PctCenter2–3, difference in time spent in the center of the open field between day 2 and day 3; TimeExploringObjs_Day1–2, total time spent with objects on day 1 and day 2 of novel object; PctTime_Novel, percent of time spent with the novel object on novel object day 2. The bold values are the percent variance for the component as a whole.*

To clarify the differences between males and females, we split sexes to run separate PCAs. Four reduction factors were identified in both sexes. In females, the first factor accounted for 28.17% of the variance, the second for 18.97%, the third for 15.75%, and the fourth for 10.83%. In males, the first factor accounted for 23.79% of the variance, the second for 18.65%, third for 15.68%, and the fourth for 11.64%. The loadings for the factors and the variables in each are indicated in Table 2.

The components of these factors were distinct in each sex. Factor 1 in females was primarily comprised of activity measures across the open field and activity monitoring, whereas factor 1 in males was comprised of open field activity and time in the center, and activity measures from activity monitoring fell into the second factor. Measures of percent time in the center of the open field loaded onto the third factor in both males and females, though novel object measures differed between the sexes. In males, the percent time spent exploring the novel object and the time spent with the objects both loaded onto factor 4, whereas these were split between factor 2 and factor 4 in females.

An ANOVA on the PCA scores did not reveal differences based on amifostine dose or radiation exposure for either sex. However, these data highlight the unique effects of radiation and amifostine in males and females and reinforce the need to include both sexes in experiments.

## Discussion

This study shows that male and female mice respond differently to combined heavy ion radiation and supports amifostine may be used as a mitigator of heavy ion radiation-induced cognitive injury in a sex-dependent fashion. Namely, radiation disrupted light activity, novel object recognition, and regional connectivity in male mice. Amifostine rescued novel object recognition, but also had its own effects on light activity and brain connectivity. Additionally, amifostine combined with radiation altered spatial habituation, object exploration, and novel object recognition in females.

The sex differences we observed in radiation susceptibility to cognitive injury is consistent with some, but not other, earlier studies. The results of the current study are in line with findings showing that male mice exhibit impaired novel object recognition 12 weeks following low-dose (<30 cGy) ^4^He ion exposure (400 MeV/n), but females did not (Parihar et al., 2020). This report also showed that males had a more pronounced CNS immune response after radiation than females, indicated by microglial activation, upregulation of Toll-like receptor 4, increased pro-inflammatory markers, and decreased hippocampal spine density. Yet, irradiated females did show a decrease in hippocampal dendritic complexity, which suggests that radiation induces distinct cellular changes in males and females (Parihar et al., 2020). Sex-dependent responses to radiation have also been seen following whole-body exposure to 50 cGy of ^56^Fe. Activity levels in APP/PS1 male mice were increased 1.5 months after exposure, which was not observed in females (Liu et al., 2019). Wild-type males also displayed a mild increase in microhemorrhages following radiation. Conversely, female APP/PS1 mice had a decrease in microglial activation and amyloid-beta levels after exposure, again highlighting sex-dependent cellular responses to radiation (Liu et al., 2019).

Male mice also showed long-term decreases in hippocampal neurogenesis 3 months after exposure to 1 Gy of ^28^Si (300 MeV/n) that was not seen in females (Whoolery et al., 2017). In addition, female WT mice were less susceptible than male mice to the negative effects of combined proton (252 MeV/n), ^4^He (249.3 MeV/n), and ^16^O (594.4 MeV/n) radiation 1.5 months after exposure, where male mice displayed increased anxiety and impaired object recognition that corresponded to hippocampal microglia activation and synapse loss (Krukowski et al., 2018). This report is in line with the sex-dependent differences in microglia activation following ^4^He ion radiation. Microglial differences are of particular interest when thinking about the molecular mechanisms underlying what appears to be female resistance to the detrimental effects of radiation.

Baseline sex differences in microglia number have been observed, where females have more than males (Villa et al., 2018). However, the increase in number of microglia does not correspond to inflammatory signals: analysis of the transcriptome of microglia in males revealed more transcription related to inflammation, whereas the transcriptome from female microglia was more related cellular regulation and was associated with neuroprotection (Villa et al., 2018). Importantly, this was independent of circulating estradiol levels. Exposure to single, highly charged particles as well as space flight have been shown to induce early ovarian failure while spermatogenesis is relatively resistant (Mishra and Luderer, 2019). Thus far, reports on the effects of multi-particle, simulated GCR exposure on sex steroid levels have not been reported.

In contrast to the results discussed above, contextual fear conditioning was impaired in female but enhanced in male WT mice 12 weeks after 300 cGy of cranial ^56^Fe irradiation (Villasana et al., 2010). We have also previous reported impairments in spatial memory in both WT male and female mice 2 weeks following 10, 20, or 50 cGy of ^56^Fe (600 MeV/n) irradiation (Haley et al., 2013). Conversely, whole-body ^56^Fe irradiation at 10, 50, or 200 cGy did not lead to sex-dependent cognitive impairments 2–8 weeks later (Pecaut et al., 2004). Moreover, no differences in hippocampus-dependent learning and memory were seen in WT mice after 10 or 50 cGy ^56^Fe irradiation when assessed 1 month later (Liu et al., 2019). These discrepancies highlight the care needed when looking at type, dose, energy, and time post-radiation. A brief break-down of the major sex-dependent behavioral findings from these studies and how they compare to our current study is illustrated in Table 3. It will be important to continue assessing the effects of mixed beam exposure at different doses, energies, and timepoints post-radiation and in the context of different genetic backgrounds in order to further clarify sex differences in radiation response for specific behavioral and cognitive performance measures.

**TABLE 3:** Brief overview of the type of radiation, dose, energy, delivery, time delay, and major sex-dependent findings from a selection of previous studies in comparison to the current study.

Beam type	Dose	Energy	Delivery	Interval	Major sex-dependent effects of radiation	References
Proton, ^28^Si, ^4^He, ^16^O, ^56^Fe	50 cGy	1000 and 250, 600, 250, 350, 600 MeV/n, respectively	Whole body	3 months	Impaired NOR and altered activity during the light period in males, not females	Current study
^4^He	<30 cGy	400 MeV/n	Whole body	4 months	Impaired NOR in males, not females	Parihar et al., 2020
^56^Fe	50 cGy	968.4 MeV/n	Whole body	1.5 months	Increased activity in APP/PS1 males, but not females	Liu et al., 2019
^28^Si	100 cGy	300 MeV/n	Whole body	3 months	Decreased hippocampal neurogenesis in males, but not females	Whoolery et al., 2017
Proton, ^4^He, ^16^O	15 and 50 cGy	525, 249.3, 594.4 MeV/n, respectively	Whole body	1.5 months	Increased measures of anxiety, impaired novel object recognition, activation of hippocampal microglia, and synapse loss in males, but not females	Krukowski et al., 2018
^56^Fe	300 cGy		Cranial	4 months	Impaired contextual fear conditioning in females, but increased contextual fear conditioning in males	Villasana et al., 2010
^56^Fe	10, 20, and 50 cGy	600 MeV/n	Whole body	2 weeks	Impaired spatial memory in both males and females	Haley et al., 2013
^56^Fe	10, 50, and 200 cGy	1055 MeV/n	Whole body	2–8 weeks	No sex-dependent cognitive impairments	Pecaut et al., 2004
^56^Fe	10 and 50 cGy	968.4 MeV/N	Whole body	1 months	No changes to hippocampus-dependent memory	Liu et al., 2019
Proton, ^4^He, ^16^O, ^28^Si, ^48^Ti, ^56^Fe	25, 50, and 200 cGy	1000, 250, 250, 263, 1000, 1000 MeV/N, respectively	Whole body	3 months	Impaired NOR in males at 25 cGy and in females at 50 and 200 cGy	Raber et al., 2020
Proton, ^16^O, ^28^Si	25, 50, and 200 cGy	1000, 250, 263 MeV/n, respectively	Whole body	3 months	Impaired NOR in both males and females at 50 and 200 cGy	Raber et al., 2019

Focusing on the more recent combined-particle experiments, recent work from our lab has shown sex-specific effects in novel object recognition 3 months after sequential six-beam exposure (50% protons at 1 GeV, 20% ^4^He ions at 250 MeV/n, 7.5% ^16^O ions at 250 MeV/n, 7.5% ^28^Si ions at 263 MeV/n, 7.5% ^48^Ti ions at 1 GeV/n, and 7.5% ^56^Fe ions at 1 GeV/n), with male B6D2F1 mice showing impaired recognition at 25 cGy and females showing impaired recognition at 50 and 200 cGy (Raber et al., 2020). Cortical BDNF levels were increased in males exposed to 50 cGy, but unchanged in females. Yet, females and males both exhibited impaired novel object recognition 3 months following 50 and 200 cGy exposure to a sequential three-beam radiation exposure (60% protons at 1 GeV, 20% ^16^O at 250 MeV/n, and 20% ^28^Si at 263 MeV/n) (Raber et al., 2019). As with exposure to six sequential beams, BDNF levels were changed in males, albeit in the opposite direction. Males exposed to 200 cGy displayed a decrease in cortical BDNF. Again, these results reinforce the care needed when assessing combined effects of different particles.

Previous studies characterizing amifostine have focused on ameliorating the negative side effects of photon radiotherapy and chemotherapy (Bogo et al., 1985; Cheema et al., 2019). In humans, patients often report side effects following high doses of amifostine (200+ mg/m^2^) that include hypotension and nausea (Rades et al., 2004) and amifostine analogs are being developed to reduce side effects (Peebles et al., 2012). Yet, there are very few reports on how amifostine might affect learning and memory. One report indicated that a dose of 214 mg/kg of amifostine 30 min prior to 200 cGy of whole-body gamma radiation was able to rescue novel object recognition the following day and hippocampal neurogenesis 12 h later in male mice (Lee et al., 2010). Notably, here we tested mice 3 months after an acute amifostine injection. Preliminary data from our lab indicated that there were potential long-term protective effects of amifostine: an acute administration of an amifostine analog was sufficient to rescue long-term hippocampus-dependent learning in males 3 months later (Bacher et al., 2019). However, we did not originally predict the long-term, independent behavioral effects of amifostine on both sexes, nor have others assessed behavioral and cognitive effects of amifostine at such extended time points.

Also of important note, all previous studies regarding the effects of amifostine on learning and memory involved only male rodents, in contrast to our current study. To the best of our knowledge, the pharmacokinetics of amifostine as it relates to sex hormones have not been reported. The sex-dependent responses to amifostine, radiation, and their combination are imperative to consider when assessing the potential for amifostine to mitigate negative radiation-induced cognitive injury. Future studies are warranted for a better understanding of the distinct effects of amifostine in males and females.

The alterations in activity during the light and dark periods in male mice following amifostine treatments and radiation exposure are also in line with previous studies. High doses (400 or 750 mg/kg) of amifostine specifically reduced locomotion during the dark cycle for ∼8 h after treatment (Srinivasan et al., 2002). Assessment of astronauts during space missions has shown disruptions to light-dark cycles and sleep/wakefulness, which can in turn affect performance (Dijk et al., 2001). Additionally, circadian rhythms appear to play a role in side effects of high-dose radiation therapy, with evening radiotherapy leading to higher levels of detrimental gastrointestinal disturbances (Hsu et al., 2016). Our observation that both amifostine and radiation increase activity during typical sleep periods indicate that timing of administration needs to be considered, as well as possibly implementing other measures to assist in maintaining physiological sleep-wake cycles.

We chose to explore how radiation and amifostine affected whole-brain cFos expression based on previous work showing that IEGs are induced following ionizing radiation, such as immediately following exposure to X-rays (0.25–0.5 Gy) (Weichselbaum et al., 1994), gamma rays (0.3 Gy) (Nishad and Ghosh, 2016), or ^137^Cs (2–25 Gy) (Hong et al., 1997). This is especially clear in the hippocampus, as whole-body irradiation with 1 Gy of ^56^Fe ions increased expression of hippocampal *Arc* after fear conditioning (Raber et al., 2013). Notably, this increase in hippocampal IEGs occurred 3 months after radiation exposure, the same timeline as this current study. While we did not find differences in overall activation, we found intriguing long-term changes in co-activation across regions, which is in line with these previous data, and parallels MRI data from humans.

MRI analysis of astronauts pre- and post-flight mission has shown narrowing of the central sulcus and an upward shift of the brain, specifically following long-term missions (Roberts et al., 2017). A case-study of an astronaut after a long-duration spaceflight revealed changes in the default mode network and resting state functional connectivity between the motor cortex and cerebellum (Demertzi et al., 2016). While we did not observe radiation-dependent changes in connectivity within the cerebellum similar to those seen in other analyzed brain regions, our data compliments the connectivity data from the astronaut, indicating that exposure to space radiation likely also has an effect on the functional communication between brain regions. Moreover, MRI studies in cancer patients have shown changes in hippocampal volume and connectivity after completing treatment regimens (Dietrich et al., 2015; Cheng et al., 2017). These highly unique and informative data provide insight into regions that may be particularly susceptible to heavy ion radiation and can be used to develop methods to monitor and treat both astronauts and cancer patients prior to, during, and after missions or treatment regimens.

In addition to the radiation-induced increase in connectivity, 107 mg/kg of amifostine by itself also increased regional co-activation in the cerebrum, brainstem, and the object-recognition specific circuit, but differently than the radiation exposure. The long-term changes in cFos co-activation caused by amifostine could be a compensatory response to the stimulus (the novel object test), though we did not observe cognitive deficits in sham-irradiated males injected with 107 mg/kg of amifostine. The difference between the radiation- and amifostine-induced increases may lie in cellular sub-type. For example, the number of GABA-ergic cells in the infralimbic cortex was decreased following contextual fear conditioning in male WT mice that received 1 Gy of post-training gamma radiation (Kugelman et al., 2016). GABA signaling to hippocampal pyramidal cells was also shown to be increased in male mice 5–9 weeks after exposure to 0.5 Gy proton irradiation (150 MeV/n) (Lee et al., 2017), further suggesting cell-type specificity of radiation-induced changes to synaptic plasticity. Currently, there is no published research regarding possible cell-type specificity of amifostine; this should be explored in future efforts.

Notably, 107 mg/kg of amifostine ameliorated the high correlations induced by radiation in the cerebrum, brainstem, cerebellum, and object-recognition circuit. The difference detected in the cerebellum is especially interesting, as the only groups detected to be different were the radiation groups with or without amifostine. While the Rad-107 mg/kg group was still different than the Sham-Saline group in most cases, these results indicate the potential for a medium dose of amifostine to mitigate the cellular effects of radiation, though deeper investigations into cell type and timing are necessary. Future efforts are warranted to further characterize the extent of amifostine’s long-term effects in both males and females following different acute and chronic proton and heavy ion radiation exposures.

### Equations

Discrimination index


$$\text{DI}=\left({\text{T}}_{N}-{\text{T}}_{F}\right)÷{\text{T}}_{T}.$$

## Author’s Note

We do not have materials that are listed in the RRID.

## Data Availability Statement

The raw data supporting the conclusions of this article will be made available by the authors, without undue reservation.

## Ethics Statement

The animal study was reviewed and approved by the OHSU IA CUC.

## Author Contributions

SB and JR were responsible for creating the hypotheses and experimental design. SB, SN, JL, and RP were all involved in experimental procedures, including radiation exposure, behavioral and cognitive testing, tissue processing, and data analysis. SB and BZ were the primary people responsible for data analysis. SB, BZ, and JR were involved in interpretation. SB wrote the manuscript. BZ, SN, JL, RP, and JR contributed to editing. All authors contributed to the article and approved the submitted version.

## Conflict of Interest

The authors declare that the research was conducted in the absence of any commercial or financial relationships that could be construed as a potential conflict of interest.

## Publisher’s Note

All claims expressed in this article are solely those of the authors and do not necessarily represent those of their affiliated organizations, or those of the publisher, the editors and the reviewers. Any product that may be evaluated in this article, or claim that may be made by its manufacturer, is not guaranteed or endorsed by the publisher.
