# Supplementary material for: Amifostine (WR-2721) Mitigates Cognitive Injury Induced by Heavy Ion Radiation in Male Mice and Alters Behavior and Brain Connectivity
Source: Front Physiol. 2021 Nov 16;12:770502. doi: 10.3389/fphys.2021.770502 (PMC8637850; doi:10.3389/fphys.2021.770502)
Supplement: Supplementary file 1 [file Data_Sheet_1.docx]

**Supplemental Material**

Amifostine (WR-2721) mitigates cognitive injury induced by heavy ion radiation in male mice and alters behavior and brain connectivity

**Supplemental Table 1.** Differences between male and female C57Bl/6J and response to radiation and amifostine in our behavioral measures.

| **Measure** | **Type of Test** | **Factors** | **Significant Finding(s) based on Sex** | ***t* or *F* statistic** | ***p* value** | **Direction of Change** |
| --- | --- | --- | --- | --- | --- | --- |
| **Body Weight** | RM ANOVA | Sex Radiation Amifostine Dose Time | Main Effect of Sex | *F*(1,84) = 1191.58 | *p* < 0.0001 | Males weighed more than Females |
|  |  |  | Radiation by Sex Interaction | *F*(1,84) = 8.217 | *p* = 0.005 |  |
| **Total Average Activity during Light** | ANOVA | Sex Radiation Amifostine Dose | Trend towards a Main Effect of Sex | *F*(1,60) = 3.840 | *p* = 0.055 | Females were more active than Males; Amifostine decreased activity in Females, but increased it in Males |
|  |  |  | Sex by Amifostine Dose Interaction | *F*(2,60) = 4.579 | *p* = 0.014 |  |
|  |  |  | Sex by Amifostine Dose by Radiation Interaction | *F*(2,60) = 3.852 | *p* = 0.027 |  |
| **Total Average Activity during Dark** | ANOVA | Sex Radiation Amifostine Dose | Main Effect of Sex | *F*(1,60) = 21.686 | *p* < 0.0001 | Females were more active than Males. |
| **Activity Ratio Dark / Light** | ANOVA | Sex Radiation Amifostine Dose | Main Effect of Sex | *F*(1,60) = 6.282 | *p* = 0.015 | Females had a higher ratio than Males; Amifostine increased this ratio in Females, but decreased it in Males. Radiation also decreased the ratio in Males. |
|  |  |  | Sex by Amifostine Dose Interaction | *F*(2,60) = 4.568 | *p* = 0.014 |  |
|  |  |  | Sex by Amifostine Dose by Radiation Interaction | *F*(2,60) = 5.836 | *p* = 0.005 |  |
| **Average Activity during Light** | RM ANOVA | Sex Radiation Amifostine Dose Time | Time by Sex Interaction | *F(*3.576,214.576) = 7.171 | *p* < 0.0001 | Females were more active than Males; Amifostine decreased activity in Females, but increased it in Males. Radiation increased activity in Females. |
|  |  |  | Time by Sex by Radiation Interaction | *F*(3.576,214.576) = 2.578 | *p* = 0.045 |  |
|  |  |  | Time by Sex by Amifostine Dose by Radiation Interaction | *F*(7.153,214.576) = 4.399 | *p* < 0.0001 |  |
|  |  |  | Sex by Amifostine Dose Interaction | *F*(2,60) = 4.536 | *p* = 0.015 |  |
|  |  |  | Sex by Amifostine Dose by Radiation Interaction | *F*(2,60) = 3.845 | *p* = 0.027 |  |
| **Average Activity during Dark** | RM ANOVA | Sex Radiation Amifostine Dose Time | Time by Sex Interaction | *F(*3.325,199.330) = 7.521 | *p* < 0.0001 | Females were more active than Males; Radiation mildly increased activity in Females, but not Males; Amifostine increased activity in Females, but not Males. |
|  |  |  | Time by Radiation Interaction | *F(*3.325,199.330) = 3.976 | *p* = 0.007 |  |
|  |  |  | Time by Sex by Radiation Interaction | *F(*3.325,199.330) = 3.831 | *p* = 0.008 |  |
|  |  |  | Time by Sex by Amifostine Dose Interaction | *F(*6.651,199.330) = 3.356 | *p* = 0.003 |  |
|  |  |  | Time by Amifostine Dose by Radiation Interaction | *F(*6.651,199.330) = 2.357 | *p* = 0.027 |  |
|  |  |  | Main Effect of Sex | *F*(1,60) = 20.243 | *p* < 0.0001 |  |
| **Open Field Total Distance Moved** | RM ANOVA | Sex Radiation Amifostine Dose Time | Main Effect of Sex | *F*(1,84) = 72.633 | *p* < 0.0001 | Females moved more than Males; Amifostine increased the change over time in Males, but not Females. |
|  |  |  | Time by Sex Interaction | *F*(1.686,141.605) = 9.89 | *p* < 0.0001 |  |
|  |  |  | Time by Sex by Amifostine Dose Interaction | *F*(3.372,141.605) = 6.695 | *p* < 0.0001 |  |
| **Open Field Average Velocity** | RM ANOVA | Sex Radiation Amifostine Dose Time | Main Effect of Sex | *F*(1,84) = 72.633 | *p* < 0.0001 | Females moved more than Males; Amifostine increased the change over time in Males, but not Females. |
|  |  |  | Time by Sex Interaction | *F*(1.686,141.597) = 9.903 | *p* < 0.0001 |  |
|  |  |  | Time by Sex by Amifostine Dose Interaction | *F*(3.372,141.597) = 6.696 | *p* < 0.0001 |  |
| **Open Field Percent Time in the Center** | RM ANOVA | Sex Radiation Amifostine Dose Time | Main Effect of Sex | *F*(1,84) = 10.887 | *p* = 0.001 | Females spent more time in the center than males |
|  |  |  | Time by Sex Interaction | *F*(2,168) = 4.059 | *p* = 0.019 |  |
|  |  |  |  |  |  |  |
| **Novel Object Time Spent Exploring Objects** | RM ANOVA | Sex Radiation Amifostine Dose Time | Trend towards Sex by Radiation by Amifostine Interaction | *F*(2,84) = 2.670 | *p* = 0.075 | Females explored the objects more than Males; Amifostine by itself increased object exploration in Females, but Amifostine + Radiation decreased it. |
|  |  |  | Trend towards Time by Sex by Radiation Interaction | *F*(1,84) = 3.669 | *p* = 0.059 |  |
| **Novel Object Discrimination Index** | ANOVA | Sex Radiation Amifostine Dose | No significant sex effects or interactions |  |  |  |

**Supplemental Table 2**. Breakdown of brain regions used for connectivity analysis of the cerebrum, brainstem, and cerebellum.

| **Abbreviation** | **Full Name** | **Category** | **Analysis** |
| --- | --- | --- | --- |
| FRP | frontal pole, cerebral cortex | Sensation/Perception | Cerebrum |
| MO | somatomotor areas | Sensation/Perception | Cerebrum |
| SS | somatosensory areas | Sensation/Perception | Cerebrum |
| PTLp | Posterior parietal association areas | Sensation/Perception | Cerebrum |
| MOB | main olfactory bulb | Sensation/Perception | Cerebrum |
| AOB | accessory olfactory bulb | Sensation/Perception | Cerebrum |
| AON | anterior olfactory nucleus | Sensation/Perception | Cerebrum |
| BA | bed nucleus of the accessory olfactory tract | Sensation/Perception | Cerebrum |
| NLOT | nucleus of the lateral olfactory tract | Sensation/Perception | Cerebrum |
| OT | olfactory tubercle | Sensation/Perception | Cerebrum |
| LS | lateral septal nucleus | Sensation/Perception | Cerebrum |
| MSC | medial septal complex | Sensation/Perception | Cerebrum |
| SF | septofimbrial nucleus | Sensation/Perception | Cerebrum |
| TRS | triangular nucleus of septum | Sensation/Perception | Cerebrum |
| PIR | piriform area | Sensation/Perception | Cerebrum |
| TR | postpiriform transition area | Sensation/Perception | Cerebrum |
| TT | taenia tecta | Sensation/Perception | Cerebrum |
| EP | endopiriform nucleus | Sensation/Perception | Cerebrum |
| VIS | visual areas | Sensation/Perception | Cerebrum |
| ORB | orbital area | Sensation/Perception | Cerebrum |
| AUD | auditory areas | Sensation/Perception | Cerebrum |
| GU | gustatory areas | Sensation/Perception | Cerebrum |
| ACA | anterior cingulate area | Fear/Anxiety | Cerebrum |
| ILA | infralimbic area | Fear/Anxiety | Cerebrum |
| DP | dorsal peduncular area | Fear/Anxiety | Cerebrum |
| PL | prelimbic area | Fear/Anxiety | Cerebrum |
| AI | agranular insular area | Fear/Anxiety | Cerebrum |
| BAC | bed nucleus of the anterior commissure | Fear/Anxiety | Cerebrum |
| BST | bed nucleus of the stria terminalis | Fear/Anxiety | Cerebrum |
| AAA | anterior amygdalar area | Fear/Anxiety | Cerebrum |
| BLA | basolateral amygdalar nucleus | Fear/Anxiety | Cerebrum |
| BMA | basomedial amygdalar nucleus | Fear/Anxiety | Cerebrum |
| LA | lateral amygdalar nucleus | Fear/Anxiety | Cerebrum |
| CEA | central amygdalar nucleus | Fear/Anxiety | Cerebrum |
| MEA | medial amygdalar nucleus | Fear/Anxiety | Cerebrum |
| PA | posterior amygdalar nucleus | Fear/Anxiety | Cerebrum |
| COA | cortical amygdalar area | Fear/Anxiety | Cerebrum |
| PAA | piriform-amygdalar area | Fear/Anxiety | Cerebrum |
| IA | intercalated amygdalar nucleus | Fear/Anxiety | Cerebrum |
| ACB | nucleus accumbens | Basal Ganglia | Cerebrum |
| CP | caudoputamen | Basal Ganglia | Cerebrum |
| FS | fundus of striatum | Basal Ganglia | Cerebrum |
| GPe | globus pallidus, external | Basal Ganglia | Cerebrum |
| GPi | globus pallidus internal | Basal Ganglia | Cerebrum |
| SI | substantia innominata | Basal Ganglia | Cerebrum |
| ECT | ectorhinal area | Spatial Memory | Cerebrum |
| PERI | perirhinal area | Spatial Memory | Cerebrum |
| HIP | hippocampal region | Spatial Memory | Cerebrum |
| RHP | retrohippocampal region | Spatial Memory | Cerebrum |
| SH | septohippocampal nucleus | Spatial Memory | Cerebrum |
| TEa | temporal association areas | Spatial Memory | Cerebrum |
| RSP | retrosplenial area | Spatial Memory | Cerebrum |
| MA | Magnocellular nucleus | Spatial Memory | Cerebrum |
| VISC | visceral area | Spatial Memory | Cerebrum |
| DORpm | Thalamus, polymodal association cortex related | Interbrain | Brainstem |
| DORsm | thalamus, sensory-motor cortex related | Interbrain | Brainstem |
| LZ | Hypothalamic lateral zone | Interbrain | Brainstem |
| MEZ | Hypothalamic Medial Zone | Interbrain | Brainstem |
| PVZ | periventricular zone | Interbrain | Brainstem |
| PVR | periventricular region | Interbrain | Brainstem |
| CUN | cuneiform nucleus | Midbrain | Brainstem |
| PRT | pretectal region | Midbrain | Brainstem |
| EW | edinger-westphal nucleus | Midbrain | Brainstem |
| MRN | midbrain reticular nucleus | Midbrain | Brainstem |
| PPN | pedunculopontine nucleus | Midbrain | Brainstem |
| PAG | periaqueductal gray | Midbrain | Brainstem |
| RAmb | midbrain raphe nucleus | Midbrain | Brainstem |
| RN | red nucleus | Midbrain | Brainstem |
| RR | midbrain reticular nucleus, retroubral area | Midbrain | Brainstem |
| IC | inferior colliculus | Midbrain | Brainstem |
| NB | Nucleus of the brachium of the inferior colliculus | Midbrain | Brainstem |
| SCm | superior colliculus, motor related | Midbrain | Brainstem |
| SCs | Superior colliculus, sensory related | Midbrain | Brainstem |
| PBG | parabigeminal nucleus | Midbrain | Brainstem |
| SNc | substantia nigra, compact part | Midbrain | Brainstem |
| SNr | substantia nigra, reticular part | Midbrain | Brainstem |
| VTA | Ventral tegmental area | Midbrain | Brainstem |
| AT | anterior tegmental nucleus | Midbrain | Brainstem |
| VTN | ventral tegmental nucleus | Midbrain | Brainstem |
| MEV | midbrain trigeminal nucleus | Midbrain | Brainstem |
| SAG | nucleus sagulum | Midbrain | Brainstem |
| LT | lateral terminal nucleus of the accessory optic tract | Midbrain | Brainstem |
| MY-mot | medulla, motor related | Hindbrain | Brainstem |
| MY-sat | medulla, behavioral state related | Hindbrain | Brainstem |
| MY-sen | medulla, sensory related | Hindbrain | Brainstem |
| P-mot | pons, motor related | Hindbrain | Brainstem |
| P-sat | pons, behavioral state related | Hindbrain | Brainstem |
| P-sen | pons, sensory related | Hindbrain | Brainstem |
| CENT | central lobule | Vermal Region | Cerebellum |
| CUL | culmen | Vermal Region | Cerebellum |
| DEC | Declive (VI) | Vermal Region | Cerebellum |
| FOTU | Folium-tuber vermis (VII) | Vermal Region | Cerebellum |
| LING | lingula (I) | Vermal Region | Cerebellum |
| NOD | Nodulus (X) | Vermal Region | Cerebellum |
| PYR | Pyramus (VIII) | Vermal Region | Cerebellum |
| UVU | uvula (IX) | Vermal Region | Cerebellum |
| AN | ansiform lobule | Hemispheric Region | Cerebellum |
| COPY | copula pyramidis | Hemispheric Region | Cerebellum |
| FL | Flocculus | Hemispheric Region | Cerebellum |
| PFL | paraflocculus | Hemispheric Region | Cerebellum |
| PRM | paramedian lobule | Hemispheric Region | Cerebellum |
| SIM | simple lobule | Hemispheric Region | Cerebellum |

**Supplemental Table 3**. Brain regions important for 24-hour novel object recognition.

| **Abbreviation** | **Full Name** | **Role** |
| --- | --- | --- |
| **OLF** | Olfactory Areas | Sensation/Perception |
| **VIS** | Visual Areas | Sensation/Perception |
| **SS** | Somatosensory Areas | Sensation/Perception |
| **MO** | Somatomotor Areas | Locomotion |
| **LA** | Lateral Amygdalar Nucleus | Fear/Anxiety |
| **BLA** | Basolateral Amygdalar Nucleus | Fear/Anxiety |
| **BMA** | Basomedial Amygdalar Nucleus | Fear/Anxiety |
| **CEA** | Central Amygdalar Nucleus | Fear/Anxiety |
| **MEA** | Medial Amygdalar Nucleus | Fear/Anxiety |
| **HIP** | Hippocampal Region | Memory |
| **PERI** | Perirhinal Area | Short-term object encoding |
| **ECT** | Ectorhinal Area | Memory, Time |
| **ENT** | Entorhinal Area | Memory |
| **TEa** | Temporal Association Areas | Memory |
| **SUB** | Subiculum | Memory support |
| **PAR** | Parasubiculum | Memory support |
| **PRE** | Presubiculum | Memory support |
| **POST** | Postsubiculum | Memory support |

**
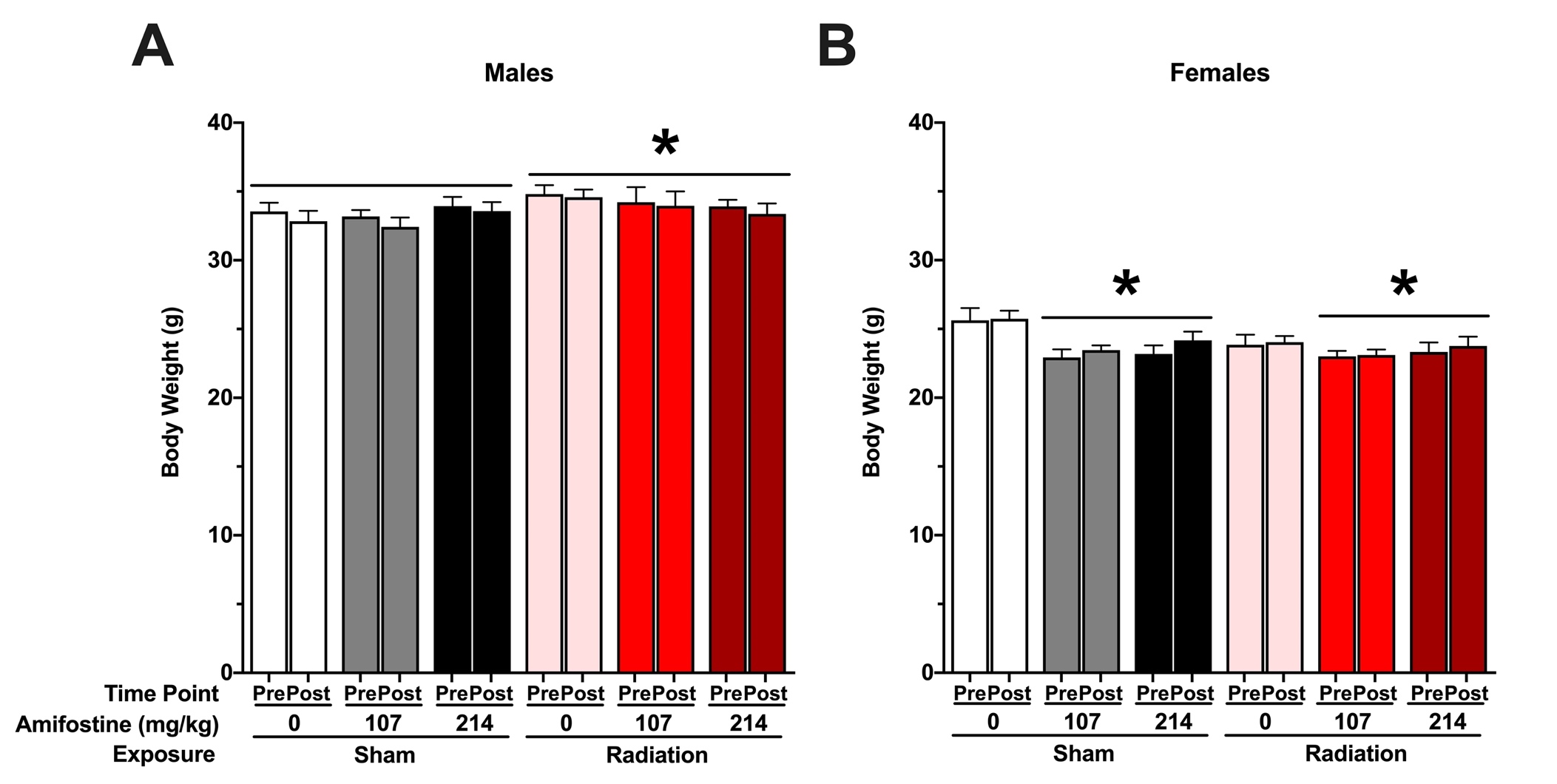
**

**Supplemental Figure 1**. Body weight before and after radiation or sham exposure with and without amifostine pre-treatment in **A)** male and **B)** female C57Bl/6J. Overall, male mice exposed to radiation weighed more (*p* < 0.05), but this did not change over time. Female mice that received amifostine weighed less (*p* < 0.05), but this also did not change over time.

**
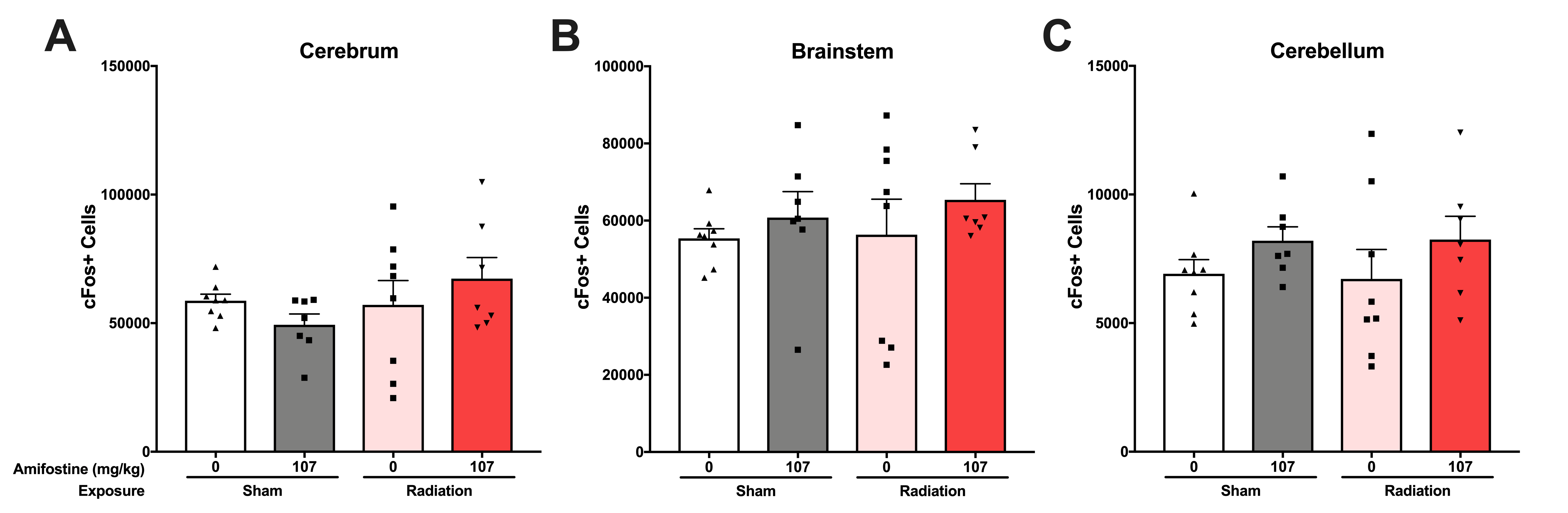
**

**Supplemental Figure 2**. The number of cFos+ cells across the whole brain. **A)** The number of cFos+ cells in the cerebrum. No significant differences were detected. **B)** The number of cFos+ cells in the brainstem. No significant differences were detected. **C)** The number of cFos+ cells in the cerebellum. No significant differences were detected.

**
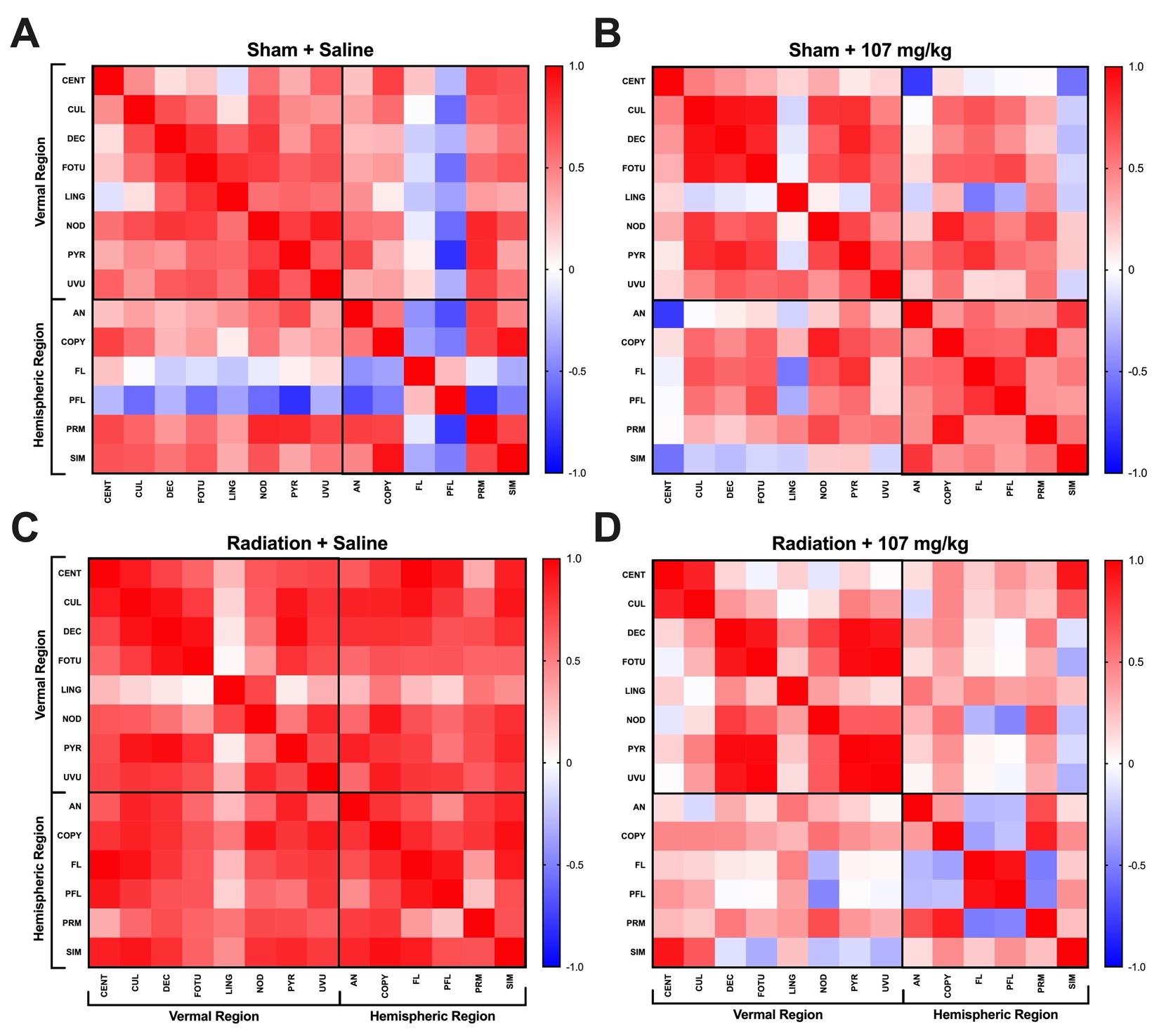
**

**Supplemental Figure 3**. cFos connectivity in the cerebellum. Pearson’s correlations were run for all regions in each distinct group: **A**) Sham-Saline, **B**) Sham-107mg/kg, **C**) Rad-Saline, **D**) Rad-107mg/kg. Bonferroni-corrected comparisons of the correlation matrices indicated that the Rad-Saline group was significantly different than the Rad-107mg/kg group only. Full names for brain regions can be seen in supplementary table 1.

**
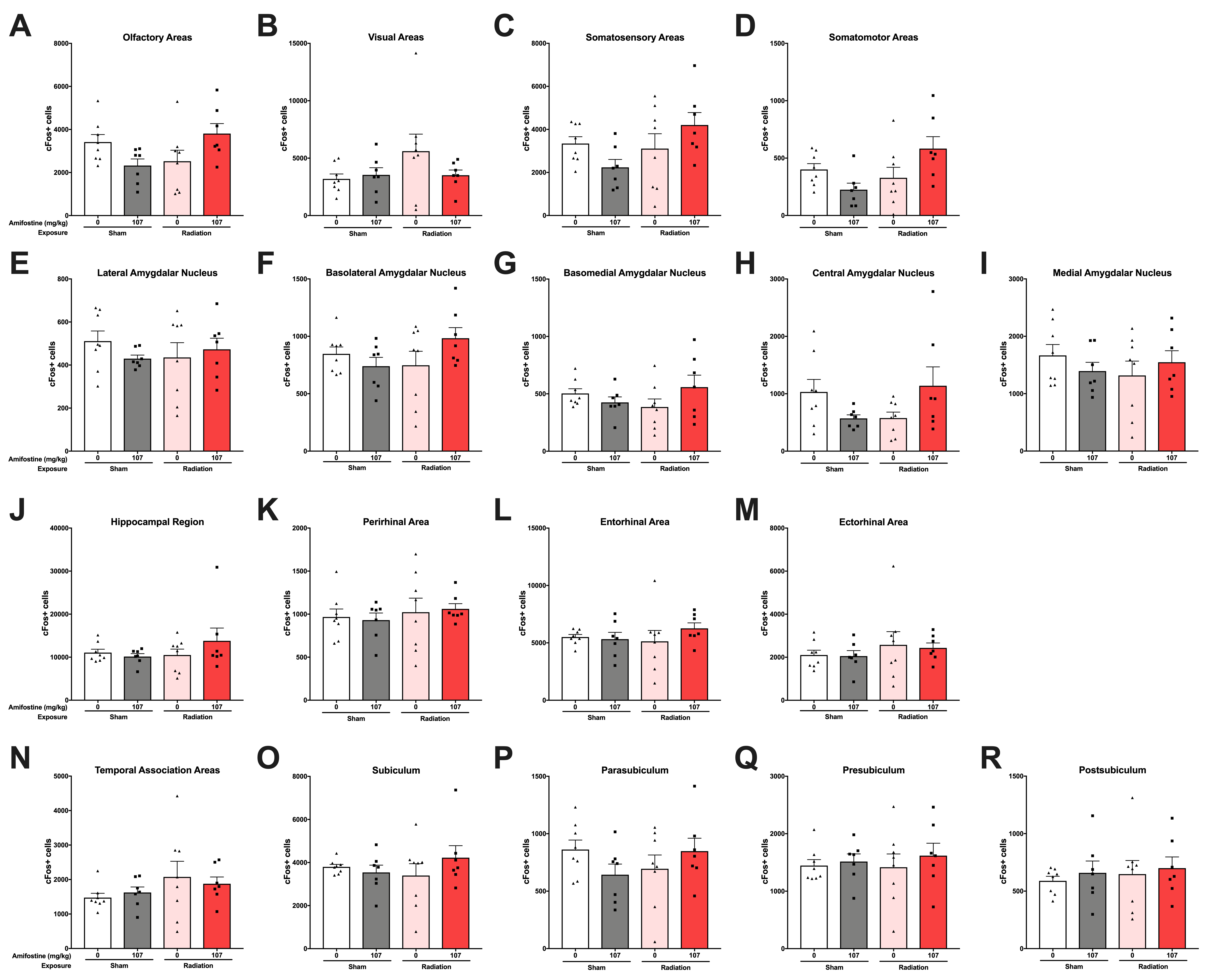
**

**Supplemental Figure 4**. The number of cFos+ cells across brain regions involved in 24-hour novel object recognition. No differences were detected between groups. *First row*: Sensory areas. **A**) Olfactory areas. **B**) Visual areas. **C**) Somatosensory areas. **D**) Somatomotor areas. *Second row:* Amygdalar nuclei. **E**) Lateral amygdalar nucleus. **F**) Basolateral amygdalar nucleus. **G**) Basomedial amygdalar nucleus. **H**) Central amygdalar nucleus. **I**) Medial amygdalar nucleus. *Third row:* Hippocampus & associated areas. **J**) Hippocampal region. **K**) Perirhinal area. **L**) Entorhinal area. **M**) Ectorhinal area. *Fourth row:* Spatial regions. **N**) Temporal association area. **O**) Subiculum. **P**) Parasubiculum. **Q**) Presubiculum. **R**) Postsubiculum.
